# Evaluation of effect of transfusion practices on infection risks in open heart surgery: Insights from a study at Amir-Al-Mominin Hospital in Golestan province, Iran, 2022

**DOI:** 10.34172/jcvtr.025.33389

**Published:** 2025-09-28

**Authors:** Seyedeh Sedigheh Hosseini, Fatemeh Tahmasebi, Mohammad Taher Hojjati, Mahdi Zahedi, Sima Besharat

**Affiliations:** ^1^Laboratory Sciences Research Center, Golestan University of Medical Sciences, Gorgan, Iran; ^2^Ischemic Disorders Research Center, Golestan University of Medical Sciences, Gorgan, Iran; ^3^Golestan Research Center of Gastroenterology and Hepatology, Golestan University of Medical Sciences, Gorgan, Iran

**Keywords:** Blood transfusion, Open heart surgery, Post-surgical infection

## Abstract

**Introduction:**

Coronary artery bypass graft surgery (CABG) is the standard treatment for obstructive coronary artery disease, particularly in patients with multi-vessel involvement or diabetes. Blood transfusions are often necessary during CABG, with rates ranging from 40% to 90%. We studied the multifactorial analysis of transfusion practices and infection risks in open heart surgery in Golestan Province, Iran.

**Methods:**

In the study we reviewed the medical records of 268 patients who underwent open heart surgery in 2022. Exclusion criteria included known immunodeficiency conditions, immunosuppressive drug use, and incomplete records. Data on risk factors (age, gender, diabetes, BMI, smoking) and laboratory results (CRP, WBC, blood cultures) were collected.

**Results:**

Out of 268 patients, 210 were analyzed (125 men, 85 women). The average ages were 57.7±9.8 for men and 58.6±9.3 for women (*P*=0.515). Diabetic patients showed a higher incidence of positive blood cultures (*P*=0.047). PC transfusion occurred in 29.5% of patients, with no significant differences between diabetic and non-diabetic groups.

**Conclusion:**

The prevalence of positive blood cultures, particularly among diabetic patients, emphasizes the importance of vigilant monitoring and management of this population to mitigate infection risks.

## Introduction

 Coronary artery bypass graft surgery (CABG) is considered the gold standard for treating obstructive coronary artery disease, particularly in patients with multi-vessel involvement or diabetes. ^1^ However, patients undergoing CABG are at risk for serious postoperative complications, including surgical wound infections and blood infections, which can lead to increased morbidity and mortality, prolonged hospital stay, and elevated healthcare costs.^2^

 Open heart surgery typically requires the highest rates of blood transfusion among all medical procedures ^3^, in which, reports indicate that 40% to 90% of patients undergoing such surgeries require blood transfusions.^4,5^ The relationship between blood transfusion and the incidence of infections following open heart surgery has been documented, identifying transfusion as a significant risk factor for postoperative infection.^6,7,8^ Consequently, we undertook this study to investigate the Multifactorial Analysis of Postoperative Transfusion Practices and Infection Risks in Open Heart Surgery at Amir-al-Mominin Hospital, a specialized cardiac center in Golestan Province, northern Iran.

## Materials and methods

 The study was conducted among patients who underwent open heart surgery in 2022 at Amir-al-Mominin Hospital. We evaluated the medical records of 268 patients who attended routine postoperative visits. Patients with known immunodeficiency diseases, those taking immunosuppressive drugs, and those with incomplete medical records (who did not returned to hospital for further monitoring) were excluded from the study.

 Necessary permissions were obtained from Golestan University of Medical Sciences, and informed consent was secured from all patients. This study was approved by the Ethics Committee of Golestan University of Medical Sciences, receiving the ethics code IR.GOUMS.REC.1400.439.

 Data on potential risk factors, including gender, age, underlying conditions (especially diabetes), BMI, and smoking status, were extracted from patient medical records. To assess the risk of infection, we performed direct clinical observations during routine visits and evaluated laboratory parameters, including high ESR, positive CRP, elevated WBC count, positive blood culture, and neutrophilila. Additionally, we assessed transfusion rate at various stages of hospitalization.

 Data analysis was conducted using SPSS version 20. Independent t-tests were used for quantitative variables, and χ^2^ tests were utilized for qualitative variables. Paired t-tests were also performed to evaluate changes in variables before and after various days after surgery.

## Results

 Out of 268 patients whose medical documents were evaluated, 210 patients were included in the analysis (125 men [59.5%] and 85 women [40.5%]). The average age of men was 57.7 ± 9.8 years, while the average age of women was 58.6 ± 9.3 years (*P *value = 0.515). As expected, the number of men with a history of smoking was significantly higher than that of women (*P* = 0.011). However, no significant differences were observed between genders in the other variables listed in Table 1.

**Table 1:** Examination of Variables According to Gender

		**Gender**		***P*** Value^**^
		**male**	**female**	
Current smoking	No	86	71	**0.011**
	Yes	39	14	
Blood culture	No Growth	116	74	0.125
	Positive culture	9	11	
Diabetic situation	Non-diabetic	84	52	0.226
	Diabetic	41	33	
PC transfusion	No	86	62	0.313
	Yes	39	23	
FFP transfusion	No	106	77	0.154
	Yes	19	8	

PC: packed cell, FFP: Fresh Frozen Plasma. ** *P* Value = T-Student statistical test

 The results of the CRP tests before surgery showed that 23 out of 125 men (18.4%) and 27 out of 85 women (31.7%) tested positive. The rate in women was significantly higher than in men (*P *value = 0.018). The average Body Mass Index (BMI) was 29.44 ± 8.6 in men and 29.4 ± 7.7 in women (*P *value = 0.979). When BMI values were divided into three groups ( < 25, 25-30, and > 30), the results indicated that no significant differences were seen among the various categories.

 The results in diabetic and non-diabetic groups also showed that the incidence of positive blood cultures differed significantly between the two groups, with the diabetic group exhibiting more than double the rate of positive cultures (*P* = 0.047) (Table 2).

**Table 2:** Table of variables in diabetic and non-diabetic groups

		**Diabetic situation**		***P*** Value**
		**Non-diabetic**	**Diabetic**	
CRP before admit	negative	109	51	0.229
	positive	27	23	
Current smoking	No	102	55	0.520
	Yes	34	19	
culture	No Growth	127	63	**0.047**
	Positive culture	9	11	
PC transfusion	No	96	52	0.541
	Yes	40	22	
FFP transfusion	No	119	64	0.496
	Yes	17	10	

PC: packed cell, FFP: Fresh Frozen Plasma. ** *P* Value = T-Student statistical test

 Additionally, pre-operative positive CRP results were significantly associated with positive blood cultures (*P *value = 0.001), while no relationship was found with other factors. However, there was no difference in CRP results after surgery concerning positive blood cultures (day 1 and day 7 were examined, with p-values of 0.150 and 0.238, respectively). Smokers had significantly higher positive CRP results at the time of admission and one day after surgery (*P* values = 0.037 and *P *values 0.013, respectively). The results also indicated that WBC counts were significantly higher in smokers (*P *value = 0.011).

 A comparison of the average hematological factors in the two groups—those with positive blood cultures (20 patients) and those with negative blood cultures (190 patients)—showed significant differences in ESR, duration of intubation, RBC count, Hb level, and WBC count after surgery (Table 3).

**Table 3:** Comparison of Average Hematological Factors in Two Groups of Positive and Negative Cultures

	**No Growth**		**Positive culture**		
	**Mean**	**Std. Deviation**	**Mean**	**Std. Deviation**	***P*** Value**
Age	57.69	9.658	61.25	8.656	0.115
BMI	29.63	8.486	27.48	5.902	0.271
ESR (millimeter/hour)	15.96	11.2	24.85	18.93	**0.049**
Duration of intubation (day)	379.6	136.688	441.32	92.736	**0.05**
Neutrophil before surgery (%)	56.346	12.3332	61.27	6.8111	0.089
WBC after surgery (/10^3^ in microliter)	14.582	4.8662	16.846	3.9207	**0.046**
RBC after surgery(/10^6^ in microliter)	4.0037	0.62157	3.6145	0.42104	**0.007**
Plt after surgery (/10^3^ in microliter)	176.95	50.773	196.7	62.873	0.108
Hb level after surgery (gr/dl)	11.113	1.4377	10.26	1.2634	**0.011**
WBC at day first (/10^3^ in microliter)	14.113	3.5958	15.03	3.2117	0.275
WBC at day 7 (/10^3^ in microliter)	11.219	3.4135	12.175	3.8539	0.241
Neutrophil at day 7 (%)	0.654	0.0804	0.672	0.0897	0.354

BMI: Body Mass Index, ESR: Erythrocyte Sedimentation Rate, WBC: White Blood Cell, RBC: Red Blood Cell, Plt: Platelet, Hb: hemoglobin, ** *P* Value = T-Student statistical test

 For patients who received packed cell (PC) transfusions, the average duration of intubation was longer than for those who did not receive blood transfusions (111.9 ± 416.09 vs. 14.1 ± 372, *P* value = 0.018), with no significant differences found in other factors. A comparison of patients who received fresh frozen plasma (FFP) versus those who did not also showed that the average duration of intubation was higher in the FFP group (134.4 ± 377.65 vs. 124.3 ± 435.96, *P *value = 0.035). The comparison of positive blood cultures between PC-transfused, FFP-transfused, and non-transfused patients showed that transfusion practice had no effect on postoperative infection.(Table 4). Additionally, no correlation was found between PC or FFP transfusion and positive blood cultures or diabetic status.

**Table 4:** Comparison of Variables in Positive Cultures between PC-Transfused, FFP-Transfused, and Non-Transfused Patients

		**PC transfusion**		**P Value**	**FFP transfusion**		***P*** Value**
		**No**	**Yes**		**No**	**Yes**	
CRP before admission	Negative	112	48	0.648	137	23	0.176
	Positive	36	14		46	4	
Current smoking	No	112	45	0.357	136	21	0.452
	Yes	36	17		47	6	
CRP in day 1 after surgery	Negative	90	38	0.429	113	15	0.305
	Positive	49	23		61	11	
Diabetic. Situation	Non-diabetic	96	40	136.000	119	17	0.358
	Diabetic	52	22		64	10	
culture	No Growth	133	57	0.429	167	23	0.244
	Positive culture	15	5		16	4	

CRP: C - reactive protein, ** *P *Value = T-Student statistical test

## Discussion

 In our study, analysis of the 210 patients undergoing open-heart surgery revealed significant findings regarding demographic variables, inflammatory markers, and clinical outcomes.

 Several inflammatory markers can be increased in patients with heart diseases. Among these, CRP appears to be significantly associated with coronary events in healthy populations, in patients with unstable angina and myocardial infarction.^9^ In our study, the comparison of two groups of patients with positive and negative blood culture showed that there was a significant difference in CRP factor before surgery. Studies have analyzed the effects of preoperative inflammatory status on the surgical outcome of patients undergoing cardiac surgery. Most of these studies showed that patients with preoperative inflammatory conditions had a higher prevalence of postoperative infections.^10^ It also showed that in the group of patients with positive culture in ESR had significant differences with the negative culture group, which verified in some studies,^11,12^ and outlined factors can highly consider as a predictive factors.

 Our findings indicated a significantly higher prevalence of smoking among male patients compared to female patients (*P* = 0.011). This aligns with previous studies that have shown a strong correlation between smoking and cardiovascular diseases, particularly in men.^13^ Smoking is known to exacerbate inflammatory responses and contribute to the pathogenesis of atherosclerosis, potentially leading to poorer surgical outcomes.^14^

 In heart surgery, the blood transfusion threshold is often considered to be hemoglobin equivalent to 7 g/dL, and this difference in reports in different centers is probably due to the difference in the selective approach regarding the blood transfusion threshold.^5^ In our study, PC transfusion was performed in 29.5% of patients, which was less than most studies. In the study of Al-Harbi et al in 2019, PC were transfused in 60.1% of patients.^15^ In the study of Tauriainen et al in 2018 on 2067 patients, red blood cells were transfused in 63.5%.^16^ In the study of Bashir et al which was conducted on 176 patients in Pakistan in 2023, PC were transfused in 43% of patients.^17^ and in China, was 37.4%. In our study, also no obvious difference was found between the diabetic and non-diabetic groups in terms of the need for PC and FFP, which was consistent with previous studies.^15,17,18^

 In our study, the prevalence of positive culture among patients was 9.5%. In studies, the prevalence of positive culture reported variable, in the study of Petti et al reported about 18%,^19^ in Copeland et al 9.8%,^20^ in Mork et al 1.7%,^21^ and in Tauriainen et al’s 8.3%.^16^ Due to the fact that the patients who only underwent isolated CABG were less exposed to direct contamination of intra-cardiac structures and artificial prostheses, they were probably at a lower risk of bloodstream infections for this reason.^16^

 In our study, 64.8% of the patients had diabetes and manifest more positive culture than non-diabetics which is consistent with the results of Zhang’s study.^22^ In studies by Badabi et al and Tauriainen et al no difference was found in the incidence of infection and positive blood culture in two diabetic and non-diabetic groups.^16,23^ According to previous studies, the frequency and severity of infections increases in people with diabetes, the reasons for which can be mentioned as defects in vascularization and disturbances in cellular immunity and phagocytic function due to hyperglycemia. Also, hyperglycemia may help the colonization and growth of organisms.^23^ so the literature suggesting that diabetes mellitus is associated with impaired immune responses; increasing susceptibility to infections post-surgery.^24^ The results highlight the need for vigilant monitoring and management of diabetic patients undergoing surgical procedures.

 While this study provides valuable insights into the relationship between demographic factors, inflammatory markers, and clinical outcomes in patients undergoing open-heart surgery, it is not without limitations. The retrospective nature of the study may introduce selection bias, and the sample size, although adequate, may limit the generalizability of the findings. Future research should focus on larger, multicenter studies to validate these findings and explore the underlying mechanisms linking diabetes, smoking, and inflammatory responses to surgical outcomes.

## Conclusion

 Our study highlights the significant relationships between demographic variables, inflammatory markers, and clinical outcomes in patients undergoing open-heart surgery. The elevated levels of inflammatory markers, particularly CRP and ESR, were associated with poorer surgical outcomes and a higher prevalence of postoperative infections. The study also revealed that a notable percentage of patients required blood transfusions, with our rates being lower than those reported in other studies, suggesting a need for further investigation into transfusion practices. Moreover, the prevalence of positive blood cultures, particularly among diabetic patients, emphasizes the importance of vigilant monitoring and management of this population to mitigate infection risks.

 Despite the valuable insights provided, the retrospective nature of the study poses limitations, necessitating further prospective studies to validate these findings and enhance our understanding of the interplay between these factors in surgical outcomes. Overall, our results advocate for a tailored approach to preoperative assessment and postoperative care, particularly for high-risk groups such as diabetic patients and smokers.

## Competing Interests

 The Authors have no conflict of interest.

## Ethical Approval

 This study was conducted following the approval of the ethics code (IR.GOUMS.REC.1400.439) by the Ethics Committee of Golestan University of Medical Sciences.
